# Biomechanical comparison of semi-rigid pediatric locking nail versus titanium elastic nails in a femur fracture model

**DOI:** 10.1007/s11832-014-0629-5

**Published:** 2014-12-16

**Authors:** Marianne Flinck, Johan von Heideken, Per-Mats Janarv, Veronica Wåtz, Jacques Riad

**Affiliations:** 1 Department of Orthopaedics, Skaraborg Hospital, Skövde, Sweden; 2 Department of Women’s and Children’s Health, Karolinska Institutet, Karolinska University Hospital, Solna, 171 77 Stockholm, Sweden; 3 Capio Artro Clinic, Stockholm, Sweden; 4 Department of Solid Mechanics, KTH Royal Institute of Technology, Stockholm, Sweden

**Keywords:** Femoral shaft fracture, Children, Flexible intramedullary nail, Biomechanics, Fracture fixation, End caps

## Abstract

**Background:**

The treatment for length-unstable diaphyseal femur fractures among school-age children is commonly intramedullary elastic nails, with or without end caps. Another possible treatment is the semi-rigid pediatric locking nail (PLN). The purpose of this biomechanical study was to assess the stability of a length-unstable oblique midshaft fracture in a synthetic femur model stabilized with different combinations of intramedullary elastic nails and with a PLN.

**Methods:**

Twenty-four femur models with an intramedullary canal diameter of 10.0 mm were used. Three groups with various combinations of titanium elastic nails (TEN) with end caps and one group with a PLN were tested. An oblique midshaft fracture was created, and the models underwent compression, rotation, flexion/extension, and a varus/valgus test, with 50 and 100 % of the forces generated during walking in corresponding planes.

**Results:**

We present the results [median (range)] from 100 % loading during walking. In axial compression, the PLN was less shortened than the combination with two 4.0-mm TEN [by 4.4 (3.4–5.4) mm vs. 5.2 (4.8–6.6) mm, respectively; *p* = 0.030]. No difference was found in shortening between the PLN and the four 3.0-mm TEN [by 7.0 (3.3–8.4) mm; *p* = 0.065]. The two 3.0-mm TEN did not withstand the maximum shortening of 10.0 mm. In external rotation, the PLN rotated 12.0° (7.0–16.4°) while the TEN models displaced more than the maximum of 20.0°. No model withstood a maximal rotation of 20.0° internal rotation. In the four-point bending test, in the coronal and the sagittal plane, all combinations except the two 3.0-mm TEN in extension withstood the maximum angulation of 20.0°.

**Conclusions:**

PLN provides the greatest stability in all planes compared to TEN models with end caps, even though the difference from the two 4.0-mm or four 3.0-mm TEN models was small.

## Background

There is no standard treatment for length-unstable diaphyseal femur fractures among school-age children [1]. The most common surgical treatment for children aged 4–14 years in our clinic is titanium elastic nails (TEN) inserted in the distal femur in a proximal direction. This stabilization is based on the principle of creating a six-point fixation using two C-shaped nails [2]. However, the technique does not always provide optimal stability, and can result in shortening, angulation, and rotation [3, 4]. From biomechanical studies and clinical experience, we have learned that end caps prevent the nails from sliding back through the insertion site, and therefore increase the axial stabilization in femur fractures [5, 6]. Another treatment option, beside external fixation and submuscular plating [7, 8], is the more recently introduced semi-rigid pediatric locking nail (PLN) [9]. The PLN is inserted through the lateral greater trochanter, avoiding the piriformis fossa, to avoid injury to the vascular supply to the proximal femoral epiphysis, which could result in avascular necrosis of the femoral head [2]. To date, there are limited reports on the semi-rigid pediatric interlocking nail regarding stability [10, 11].

The biomechanical properties of femur shaft fractures in children have been studied by several investigators, beginning in 2001 with Lee et al. (Table 1) [5, 12–25]. These studies cannot be directly compared with each other, however, because they differ regarding the type of implant, fracture, and mechanical test when considering the direction and force applied to the specimen. To our knowledge, no biomechanical or clinical data have been published comparing TEN with end caps and PLN, or studying the possibility of enhancing the stability of a fracture using four TEN instead of two for femur shaft fractures. Furthermore, there are no reports, to our knowledge, on studies where clinically relevant forces obtained from three-dimensional gait analysis have been applied to the different models tested.

**Table 1:** Summary of previous biomechanical studies on femur shaft fractures

Author (year)	Sample size^a^	Implant^b^	Fracture^c^	Biomechanical test^d^
Lee et al. [12]	5	EN	C, T	AC, R
Gwyn et al. [13]	25	TEN	B, C, O, S, T	R
Fricka et al. [14]	10	TEN	C, T	AC, R
Mahar et al. [15]	10	TEN, SEN	C, T	AC, R
Green et al. [16]	6	TEN	T	AC, R, S
Mani et al. [17]	8	TEN, EN, EF	B, T	AC, C, R, S
Mehlman et al. [18]	10	TEN	T	C, R
Goodwin et al. [19]	20	TEN	C	AC, R
Li et al. [20]	10	TEN	T	C, S
Kaiser et al. [21]	16	TEN with EC	S	AC, C, R, S
Kaiser et al. [22]	24	TEN, SEN	S	AC, C, R, S
Doser et al. [23]	20	TEN	T	C, S
Kaiser et al. [24]	24	SEN	S	AC, C, R, S
Volpon et al. [5]	9	TEN with EC	T	AC, R, S
Porter et al. [25]	50	TEN, P	C, O	AC, R

Studies are included in the Table if they used synthetic pediatric-sized femur models

^a^Total number of femur models included in the analysis

^b^Type of implant for fixation: ender nails (EN), titanium elastic nails (TEN), stainless steel elastic nail (SEN), end caps (EC), external fixator (EF), locking compression plate (P)

^c^Type of fracture: butterfly (B), comminuted (C), oblique (O), spiral (S), transverse (T)

^d^Type of biomechanical test: axial compression (AC), coronal bending (C), rotation (R), sagittal bending (S)

The hypothesis was that there is no difference regarding rotational stability, risk of shortening and bending in both the sagittal and coronal plane between the PLN and the TEN with end caps in an oblique unstable femur shaft fracture during physiological loading.

## Materials and methods

Twenty-four synthetic composite pediatric-sized femur models (fourth generation; Sawbones, Pacific Research Laboratories, Inc., Vashon, WA, USA) were used for mechanical testing. In previous biomechanical studies on femur shaft fractures in children, these pediatric synthetic models have been proven to appropriately represent biomechanical properties of human femurs [5, 12–25].

**Fig. 1:**
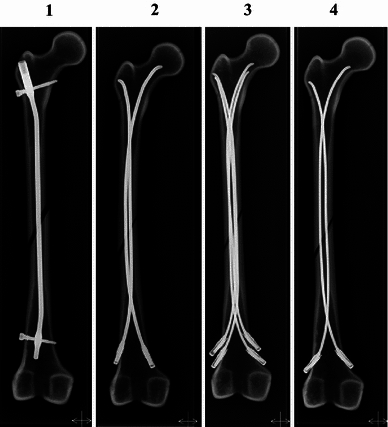
Frontal radiographs of femur models after fixation. **1** One 5.5-mm pediatric locking nail (PLN), **2** two 4.0-mm titanium elastic nail (TEN) with end cap, **3** four 3.0 mm TEN with end caps, **4** two 3.0 mm TEN with end caps

The femur models were 37.5 cm long with an intramedullary canal diameter of 10.0 mm, and were divided into four groups with six femur models in each group. Group one received a 5.5-mm semi-rigid PLN with 8.5-mm proximal geometry and distal bulb (Biomet, Parsippany, NJ, USA). Three groups received three different combinations of TEN with end caps (Synthes, Paoli, PA, USA). Group two received two nails with a diameter of 4.0 mm and group three received four nails with a diameter of 3.0 mm. The last (fourth) group received two nails with a diameter of 3.0 mm (Fig. 1).

**Fig. 2:**
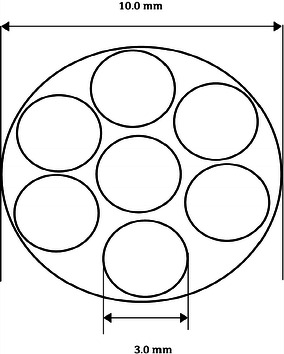
Illustration of how seven circles (elastic nails) with a diameter of 3.0 mm fit into a larger circle (medullary canal) with a diameter of 10.0 mm

Theoretically, several (seven) 3.0-mm nails fit in a canal with an inner diameter of 10 mm; however, closer to a clinical situation, we applied a maximum of four nails to fit inside a femur model with a 10.0-mm intramedullary canal (Fig. 2) [26]. It did not require any significant extra force to insert four nails, compared to two, in the femur models. All femur models were assessed with radiographs to ensure correct nail placement and there was no destruction of the intramedullary canal or splitting of the cortex.

An oblique midshaft fracture was created with a handheld saw at a 60° angle to the longitudinal axis of the shaft, and the femur models were fixed in a mold to provide the same angle of fracture in all the femur models and minimize the variability between the specimens. Sink et al. defined this fracture as length-unstable because the length of the obliquity is more than twice the diameter of the femur at the level of the fracture [3].

### Mechanical tests

The goal was to test elastic deformation of femur models to a point that would be of clinical interest, and we therefore used loads based on three-dimensional gait analysis. The gait analysis was performed with a motion analysis video capture system, and all the data were reduced using Orthotrak with the Cleveland Clinic marker set (Motion Analysis Corp., Santa Rosa, CA, USA). Standard lower-extremity joint kinematics was collected. The kinetic data was collected using two force plates (Advanced Mechanical Technology Inc. AMTI, Watertown, MA, US). From a group of five typically developed children, with a mean weight of 40 kg, three trials from each lower extremity were collected during walking at a self-selected speed. The ground reaction force vectors were collected together with the kinematic data. The maximum force during the gait cycle and the maximal external moment (N.m) were calculated from the knee joint in three planes in addition to the vertical compression force (N) [27]. The distance from the knee to the site of the fracture was small, and we considered the additional moment of inertia produced from the distal femur and thigh to be negligible. In addition there was no clear difference in the pattern of the forces during the gait cycle depending on the patient’s body weight or height, and therefore the force data were not normalized [27].

Biomechanical testing for axial compression and axial rotation was performed with a material testing machine MTS 160 kN/1100 N.m with Instron 8580+ control unit. The four-point bending test was performed using the testing device MTS 100 kN with Instron 8500 control unit. The test consisted of a load–displacement cycle. Different strain rates were evaluated in pilot tests, and there were no relevant differences. A strain rate of 0.07 mm/s was chosen. The test consisted of a preload of 50 N followed by four load–displacement cycles at 50 and 100 % of the load calculated from the gait analysis.

If the first specimen tested at 50 or 100 % was considered a failure, the following two specimens always failed. Therefore the three final specimens were not tested and the whole group was considered to be a failure and not included in the statistical model. If the first specimen did not fail, none of the following specimens failed. The groups that failed a mechanical test at 50 % for the first three models were not tested at 100 %. The groups that failed a mechanical test at 100 % for the first three models were considered to be failures and were not included in the statistical analysis.

The definition of failure was more than 10.0 mm of shortening during the test, which is in line with the radiological findings after stabilization according to the Flynn score for evaluating shortening after treatment of femur shaft fractures [27]. Failure was also considered to be more than 20.0° of rotation or angulation during the tests [5].

**Fig. 3:**
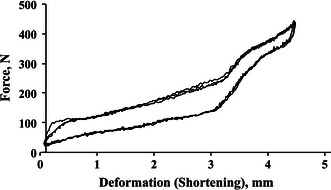
Axial compression load (N) versus deformation (mm) for fixation with PLN of an oblique femur shaft fracture at 100 % loading calculated from gait analysis during walking (after a preload of 50 N)

In each cycle, the predetermined load was reached and immediately unloaded and the next cycle was started as soon the specimen had returned to its original configuration. The fourth cycle was evaluated (Fig. 3). To simulate the normal load line in the human femur, compression force was applied through the mechanical axis, that is, from the center of the femoral head to a point centered between the femoral condyles.

Rotation was measured by the testing machine, while angulation was calculated based on the position of the loading and supporting pins of the four-point bending machine. Regardless of failure or not, each specimen always regained its original configuration, i.e. the deformation was considered to be elastic. In view of this, it was appropriate to test each specimen in all six directions.

Six stabilized femur models from each group underwent an axial compression test, an axial rotation test, and a four-point bending test in both the sagittal and coronal planes. The mechanical test was performed in four steps.

First, a compression test was performed with the femur model in an upright position. A preload of 50 N was applied and was followed by a vertical compression force of 215 N (50 % maximal force) in the first set-up and 425 N (100 % of maximal force) in the second set-up. The maximum displacement was set at 10.0 mm.

In the second step, all femurs were tested in external and internal rotation (proximal femur fracture segment relative to distal segment) with corresponding 50 and 100 % of maximal force, to a moment of 3.5 N.m in the first set-up and to a maximum torque of 7 N.m in the second setup. The femur models were tested up to a maximal 20.0° of rotation.

In the third step, a four-point bending test was performed in the coronal plane (varus/valgus). According to the gait analysis, the varus force at 100 % was only 1.5 N.m, and hence the 50 % force was very low. In valgus, the 100 and 50 % force was 7.0 and 3.5 N.m, respectively. Because of the low forces in varus, the set-up was modified and the model was tested at only 3.5 N.m in varus. In valgus, the models were tested at 3.5 N.m in the first set-up and to a maximal moment of 7 N.m in the second setup, according to the gait analysis data.

In the fourth and final step, a four-point bending test was performed in the sagittal plane (flexion/extension) to a moment of 11 N.m in the first set-up and to a maximal moment of 22 N.m in the second set-up.

### Statistical analysis

Descriptive statistics such as median and range were calculated and presented. The Mann–Whitney *U* test was used to compare continuous variables between the various TEN groups and the PLN group. *p* values less than 0.05 were considered significant. Statistical analysis was performed with SPSS (version 20, SPSS Inc., Chicago, IL, USA).

## Results

In total, 24 femur models were stabilized with different osteosyntheses and tested. One femur model, stabilized with two TEN with a diameter of 4.0 mm, broke during the test and was therefore excluded from the statistical analysis. The PLN model was set as standard because it revealed the least displacement of the four different models. The results [median (range)] of the tests loaded with 100 % force from the gait analysis are presented for displacement (Table 2).

**Table 2:** Result of the biomechanics test at 100 % loading

Test	One PLN 5.5 mm (*n* = 6)	Two TEN 4.0 mm (*n* = 5*)	*p*	Four TEN 3.0 mm (*n* = 6)	*p*	Two TEN 3.0 mm (*n* = 6)	*p*
	Median (range)	Median (range)		Median (range)		Median (range)	
Axial shortening (mm)	4.5 (3.4–5.4)	5.2 (4.8–6.6)	**0.030**	7.0 (3.3–8.4)	0.065	Failed	–
External rotation (°)	11.7 (7.0–16.4)	Failed	–	Failed	–	Not tested	–
Internal rotation (°)	Failed	Not tested	–	Not tested	–	Not tested	–
Varus angulation (°)	1.3 (0.9–2.0)	1.5 (1.2–1.9)	0.329	1.5 (1.1–2.1)	0.240	3.7 (2.2–4.9)	**0.002**
Valgus angulation (°)	1.9 (1.9–2.7)	2.3 (1.4–3.0)	1.000	4.0 (3.2–4.4)	**0.002**	6.0 (3.8–7.3)	**0.002**
Flexion (°)	2.3 (2.0–2.5)	2.8 (2.1–3.4)	0.052	3.6 (2.9–4.0)	**0.002**	6.0 (4.6–7.5)	**0.002**
Extension (°)	6.1 (4.5–6.4)	6.5 (5.3–8.1)	0.082	8.3 (6.0–9.1)	0.065	Failed	–

Comparison of deformation: the PLN was set as the reference.

*Failed*  Three models exceeded the preset limits (>10 mm or >20°). *Not tested*  Three models exceeded the preset limits (>10 mm or >20°) at 50 % force and therefore not tested at 100 % force

* One femur model broke during the test and was therefore excluded from the statistical analysis

Significant values are shown in *bold type*

In *axial compression*, the PLN model was less shortened than the combination of two 4.0-mm TEN [4.5 (3.4–5.4) mm vs. 5.2 (4.8–6.6) mm; *p* = 0.030]. No statistical difference was found in shortening between the PLN model and the model stabilized with four 3.0-mm TEN [7.0 (3.3–8.4) mm; *p* = 0.065]. The displacement was more than 10.0 mm for the models stabilized with two 3.0-mm TEN, and they were therefore considered failures according to the previously mentioned definition.

In *external rotation* the PLN provided enough stability 11.7° (7.0–16.4°), while the TEN models displaced more than the preset maximum of 20.0°. No model withstood the maximum rotation of 20.0° in *internal* rotation, when tested for the calculated force 7 N.m.

In the four-point bending test, regardless of whether the test was performed in *varus*/*valgus* or *flexion*/*extension*, all model combinations except the two 3.0-mm TEN in flexion withstood the preset maximum angulation of 20.0°. When the models were tested for varus, there was no statistical difference between the PLN and the two 4.0-mm TEN or four 3.0-mm TEN. However, there was a significant difference (*p* = 0.002) between the PLN [1.3° (0.9–2.0°)] and the two 3.0-mm TEN [3.7° (2.2–4.9°)], although the difference was small. In the valgus test, the PLN and the two 4.0-mm TEN showed no statistical difference, whereas there was a difference between the PLN and the four 3.0-mm TEN (*p* = 0.002) and between the PLN and the two 3.0-mm TEN (*p* = 0.002). In the sagittal plane, in the four-point bending test for flexion, the PLN did not show greater stability than the two 4.0-mm TEN [2.3° (2.0–2.5°) vs. 2.8° (2.1–3.4°) (*p* = 0.052)]. The flexion test comparing the PLN and the four 3.0-mm TEN revealed less stability for the TEN group than for the PLN (*p* = 0.002). Finally, the extension test showed no statistical difference between the models, and, as stated above, the two 3.0-mm TEN failed the test (Table 2).

## Discussion

The results from our biomechanical study revealed that the PLN provided the greatest stability overall, when forces corresponding to those developed during walking were applied. The combination with two 4.0-mm intramedullary elastic nails (TEN) and with four 3.0-mm TEN, with end caps, also provided high stability, except for rotation, whereas femur models stabilized with two 3.0-mm TEN failed several tests.

To our knowledge, the mechanical properties of PLN have not been analyzed previously. The present study illustrates that the PLN provides good stability to resist the physiological loading corresponding to normal walking, except for internal rotation. We noted in external rotation a trend toward increased stability for the PLN compared to the 4.0-mm combinations of TEN.

The PLN carries a potential risk of avascular necrosis of the femoral head (AVN), thinning of the femoral neck, and coxa valga [28]. However, these complications are reported in studies using the greater trochanter or piriformis fossa as entry site for the nail. Most probably, several variables play a role in a good result with the PLN; among them are the surgeon’s experience in intramedullary fixation in general, the possibility of imaging in two planes in the operating room, and the use of a traction table, to mention some; all facilitate the technique and therefore decrease the risk of complications.

Intramedullary fixation with TEN has become a popular method of treatment for pediatric femur shaft fractures. However, it has been shown that TEN is associated with more complications when used in long oblique or comminuted fracture patterns among older children compared to length-stable femur shaft fractures [3]. Clinically, varus angulation is the most important complication after a pediatric femoral shaft fracture stabilized with TEN, but both valgus and angulation in the sagittal plane have been reported [29]. Our results regarding varus deformity reveals minimal displacement and insignificant differences between the PLN, the two 4.0-mm TEN, and the four 3.0-mm TEN. Valgus, flexion, and extension displacement, on the other hand, showed increased instability comparing the different constructs with the PLN. Other complications after femur shaft fractures treated with TEN are rotational malunion (especially external rotation) and limb-length discrepancy [4]. The most commonly reported complication related to TEN, however, involves nail prominence and irritation at the nail entry site [29].

The role of the end cap is to prevent nail migration, which may prevent soft-tissue irritation and leg shortening. Furthermore, the end caps might also simplify implant removal [30]. However, biomechanical data is inconclusive regarding the contribution of the end caps to the stability of a pediatric femur shaft fracture. A biomechanical study by Volpon and colleagues on distal femur fractures indicates that end caps fitted to elastic nails may add to the stability of distal femur fracture fixation [5]. This is in contrast to a biomechanical study by Kaiser and colleagues, who could not find any improvement in the stability of a specimen with a midshaft spiral fracture stabilized with TEN and end caps [22]. Nectoux and colleagues supported the use of end caps for length-unstable fractures based on the results in a small case series on tibia and femur shaft fractures in children [30]. It was not the intent of our study to evaluate whether or not end caps improved the stability in our fracture model. We chose to use end caps in our model because even though end caps may or may not add axial or torsional stability to the fracture, it is the manufacturer’s recommendation to use end caps in length-unstable fractures [31].

One clinical study has compared semi-rigid trochanteric entry nailing with flexible nailing for treatment of length-stable femoral shaft fractures in a cohort of heavier children (47–85 kg), and they concluded that the use of TEN resulted in decreased time in the operating room, estimated blood loss, and fewer implant-related problems [32].

Inappropriate TEN sizes have been related to femoral malunion [33]. The nail diameter should correspond to between 33 % and 40 % of the narrowest medullary space diameter, and, for children 9–14 years old, the manufacturer’s recommendation is to use a 3.5- or 4.0-mm TEN [34]. The femur models used in the present study had a canal diameter of 10.0 mm, which makes the two 3.0 mm TEN too thin, which is supported by the results of our study. As expected, our results indicate that four 3.0 mm TEN provide much better stability than two 3.0 mm nails. The number of TEN used for the fracture fixation have been compared by Kanthimathi and colleagues, who found no advantage to using three instead of two nails [35]. This is in contrast to our experience from the last 15 years in our clinic, where, based on the technique described by Ender, we have used four TEN in an attempt to fill the medullary canal when two TEN nails have not provided enough stability for comminuted and unstable femur shaft fractures [36]. The rationale for adding two more nails instead of switching to thicker nails is that we believe they are easier to insert and achieve optimal fracture reduction with 3.0 mm TEN than with the stiffer 4.0 mm TEN. Regarding the size of the PLN, the manufacturer recommends a 5.5 mm nail for patients less than 45 kg and 6.5 mm for those up to 84 kg.

We recognize several potential limitations in our study. The use of synthetic bone does not provide the same stabilization of soft tissues, including the periosteum, which provide not only stability but also help in the reduction of the fracture. Another limitation regarding synthetic bone is that it does not provide normal medullary canal properties. However, in other aspects the synthetic bone corresponds to the structural properties of human bone [37]. In addition, we used a small sample size, nevertheless comparable to previous biomechanical studies that have also used similar synthetic bones (Table 1) [5, 12–25]. Furthermore, our study does not report how much load is needed to create a plastic (permanent) deformity of PLN and TEN. In external rotation, the TEN models displaced more than the PLN. However, rotational alignment is difficult to assess, both during and after surgery, and is maybe more of a problem in transverse fractures. Finally, by separating direction and force applied in the mechanical tests of the specimens we are not truly depicting the clinical situation. Unfortunately, there is no commonly used standard for the mechanical tests, which makes it difficult to compare the present results with the outcome of previous studies.

## Conclusion

Our study indicates that the PLN results in a biomechanically more stable construct than the TEN when treating length-unstable oblique femur fractures. The present biomechanical study does not, from a clinical perspective, allow any far-reaching conclusions. The differences between the tested configurations that withstood the test are small, and whether they can be duplicated clinically and their possible relevance is not known. However, we speculate that the increased stability could mean a faster and a less painful rehabilitation and a possible better outcome. The increased stability in rotation also could be of note when the osteosynthesis does not provided enough stability, important when performing derotational osteotomies. In addition, adding two more nails during the stabilization of a fracture with TEN remains an alternative.

It would be of value if the present biomechanical study on treatment of femoral shaft fractures in children were complemented by clinical outcome studies.

## Abbreviations

TEN: Titanium elastic nail
PLN: Pediatric locking nail
